# Water-Assisted Synthesis of Molybdenum Disulfide Film with Single Organic Liquid Precursor

**DOI:** 10.1038/s41598-017-02228-8

**Published:** 2017-05-16

**Authors:** Soo Ho Choi, Boandoh Stephen, Ji-Hoon Park, Joo Song Lee, Soo Min Kim, Woochul Yang, Ki Kang Kim

**Affiliations:** 1 Department of Physics, Dongguk University, Seoul, 04620 Republic of Korea; 2 Department of Energy and Materials Engineering, Dongguk University, Seoul, 04620 Republic of Korea; 3 Center for Integrated Nanostructure Physics, Institute for Basic Science (IBS), Suwon, 16419 Republic of Korea; 4 Sungkyunkwan University (SKKU), Suwon, 16419 Republic of Korea; 5 Institute of Advanced Composite Materials, Korea Institute of Science and Technology (KIST), Wanju-Gun, 55324 Republic of Korea

## Abstract

We report on the synthesis of large-area molybdenum disulfide (MoS_2_) film on an insulating substrate by means of chemical vapor deposition. A single mixture of molybdenum hexacarbonyl (Mo(CO)_6_) and dimethyl disulfide (C_2_H_6_S_2_) was utilized as an organic liquid precursor for the synthesis of MoS_2_ film. Carbon impurities stemming from the dissociation of the organic precursor are effectively removed by water oxidation, and hydrogen gas, which is a by-product of the oxidation of carbon impurities, inhibits the formation of molybdenum oxides. The use of a liquid precursor assisted with water oxidation ensures high reproducibility and full-coverage of MoS_2_ film for large area, which is not typically achieved with solid precursors such as molybdenum oxide and sulfur powder. We believe that our approach will advance the synthesis of transition metal dichalcogenides.

## Introduction

Two dimensional (2D) semiconducting transition metal dichalcogenides (s-TMdCs) MX_2_ (M = Mo or W; X = S or Se) have been highlighted due to their unique physical and chemical properties^1–6^. The energy band gaps of s-TMdCs vary from 1 to 2 eV, depending on their constituents. Monolayer s-TMdCs are the direct band gap semiconductors, and multilayer s-TMdCs are the indirect band gap semiconductors, so the unique properties of these materials can be tailored for specific applications^7–9^. For instance, monolayer s-TMdCs are very useful in optoelectronic devices due to their high photoluminescence (PL) quantum yield while multilayer s-TMdCs are more appropriate for use in high-speed thin film transistors due to their multichannel carrier path^10, 11^. Among s-TMdCs family, MoS_2_ has been extensively used as a lubricant and as an efficient catalyst for hydrogen evolution^12–14^. In particular, monolayer or few layer MoS_2_ field effect transistors (FETs) exhibit the highest carrier mobility up to 200 cm^2^ V^−1^ s^−1^ at room temperature due to the lower effective mass when compared to that of other s-TMdCs, and MoS_2_ has also been applied in flexible and transparent electronics^1, 15–17^. However, it is still challenging to obtain the large-area, high-quality MoS_2_ films.

Several methods have been suggested to obtain large-area MoS_2_ films, including chemical vapor deposition (CVD), atomic layer deposition (ALD), and molecular beam epitaxy (MBE)^18–27^. Although the thickness of MoS_2_ film can be controlled with high coverage via ALD, the crystallinity of MoS_2_ film is poor^19^. The possibility of synthesizing MoS_2_ using MBE on graphene substrate has also been proposed^18^. However, the MBE system is limited to industrial applications due to its high cost and low throughput. Compared to other methods, CVD has advantages in terms of its low cost, high throughput, and ability to grow large-area, high-quality MoS_2_ films.

Monolayer MoS_2_ was successfully synthesized via CVD in 2012, and since then, many researchers have investigated the use of various precursors, growth substrates, and growth parameters, including the pressure, flux of precursor, and temperature, to obtain large-area, high-quality MoS_2_ films^22–25^. Various seeding promoter to increase the adhesion between precursors and substrate has also been studied^20, 21^. Here, we focus on the precursors because precursors are inevitable factor to grow MoS_2_ films. Typically, solid-phase precursors such as molybdenum oxide and sulfur powders have been used^21, 26, 27^. However, it is difficult to control the vaporization of solid precursor by temperature, resulting in limitations in a consecutive and constant supply of precursors during the growth process^24, 26^. As a consequence, the growth results are not highly repeatable, and the thickness and coverage uniformities of MoS_2_ film cannot be ensured. Unlike solid-phase precursors, a gas phase precursor has an advantage in that it offers controllability^28^. Hydrogen sulfide can be utilized as sulfur precursor, but it is very toxic, and special care is therefore necessary^28^. Unfortunately, to the best of our knowledge, a gas phase precursor for molybdenum has not yet been reported. On the other hand, a liquid phase precursor is also an alternative. Metal organic chemical vapor deposition (MOCVD) with a bubbler system is widely used to grow III-nitride materials such as GaN and AlN using liquid precursors^29, 30^. Recently, a combination of diethyl sulfide ((C_2_H_5_)_2_S, liquid phase) and molybdenum hexacarbonyl (Mo(CO)_6_, solid phase) was used to grow a monolayer MoS_2_ film^31^. However, the growth time for the complete monolayer MoS_2_ film took around a day, so it is still necessary to investigate new types of precursors.

Herein, we report on the use of a single organic liquid precursor in the synthesis of large-area MoS_2_ film. The single liquid precursor is prepared by the dissolution of molybdenum hexacarbonyl in dimethyl disulfide ((CH_3_)_2_S_2_). The coverage of MoS_2_ film is controlled by adjusting the growth time, resulting in the formation of full-coverage MoS_2_ films within 15 minutes. Carbon impurities stemming from the dissociation of organic precursors are effectively removed by water oxidation, as confirmed via Raman spectroscopy and photoluminescence (PL) measurements. Furthermore, the detailed growth mechanism is discussed.

## Results and Discussion

**Figure 1:**
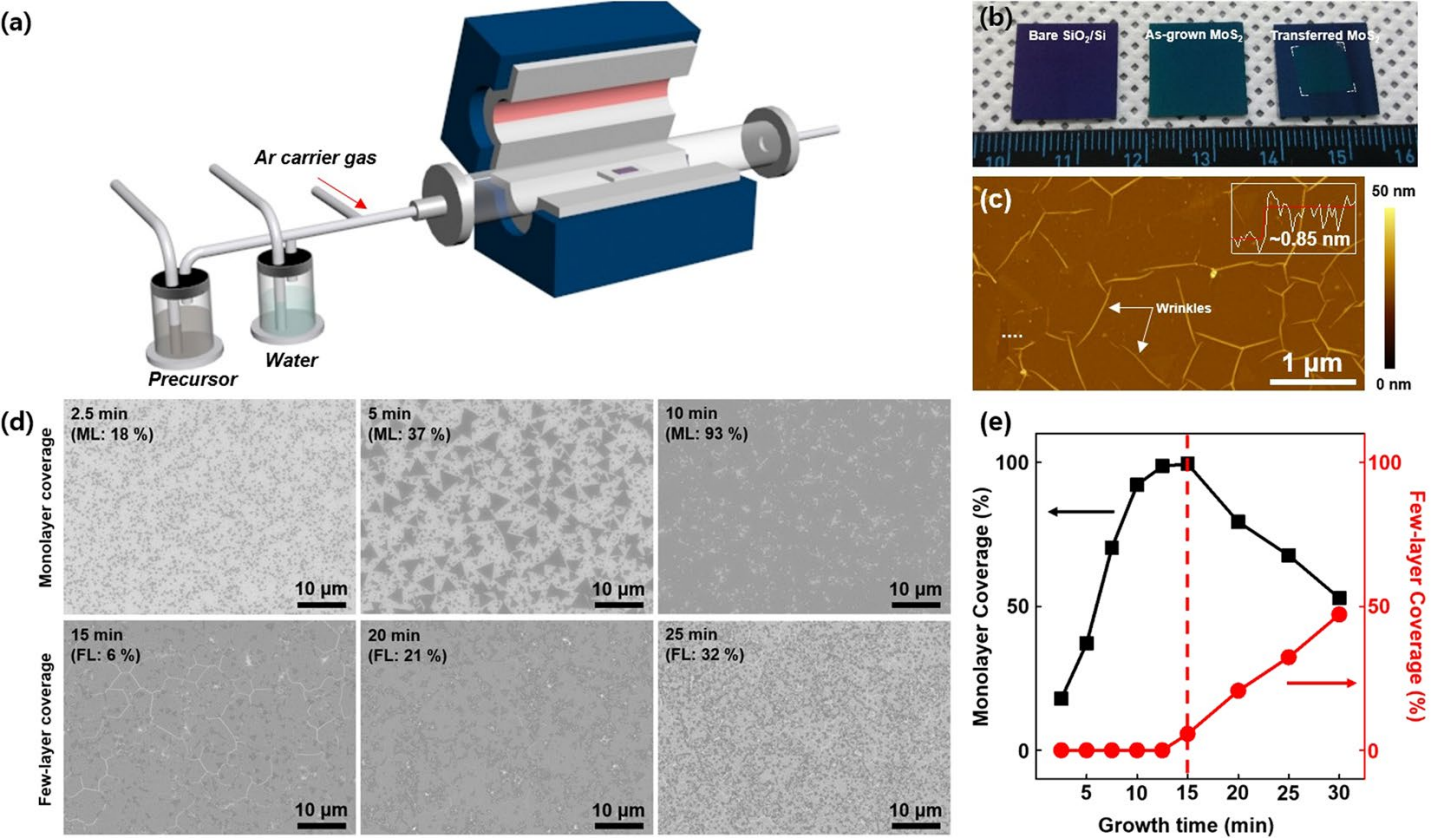
Synthesis of MoS_2_ film. (**a**) Schematic illustration of the CVD system with a liquid precursor and water bubblers. The bubblers are connected with individual mass flow controllers. (**b**) Photographs of the bare SiO_2_/Si substrate, as-grown MoS_2_ film, and transferred MoS_2_ film. (**c**) Atomic force microscopy image of the transferred MoS_2_ film. The inset indicates the height profile along the dotted white line. The white arrows present the MoS_2_ wrinkles. (**d**) SEM images of as-grown MoS_2_ as a function of the growth time. The dark region indicates MoS_2_, and the white background indicates the SiO_2_/Si substrate. The coverage for monolayer (ML) and few-layer (FL) MoS_2_ is displayed in each SEM image. (**e**) The coverage for monolayer (black) and few-layer (red) MoS_2_ regions as a function of the growth time.

The bubbler system was equipped in CVD as shown in Fig. 1(a), to synthesize the MoS_2_ film. 0.04 M of Mo(CO)_6_ powder was dissolved in (CH_3_)_2_S_2_, and the precursor solution was further analyzed via liquid chromatography-mass spectrometry (see Figure S1 in Supplementary Information). The presence of Mo and S chemicals was confirmed as Mo ions, C_2_H_8_OS, Mo(CO)_2_, Mo(CO)_5_, C_5_HMoO_6_, and C_6_HMoO_7_. Unfortunately, dimethyl disulfide was not detected due to the detection limit, but we assume that dimethyl disulfide should also be present in the precursor solution. To remove the carbon impurities, a separate water bubbler is installed. Argon is used as a carrier, and the adhesion between the precursor and the SiO_2_/Si substrate is increased by coating perylene-3,4,9,10-tetracarboxylic acid tetrapotassium salt (PTAS) on the SiO_2_/Si substrate^20^. Figure 1(b) shows photographs of bare SiO_2_/Si, as-grown MoS_2_ film, and transferred MoS_2_ film on SiO_2_/Si substrate. The color of SiO_2_/Si substrate changed violet to blue-green after growth. Furthermore, the MoS_2_ film was transferred on the SiO_2_/Si substrate using the conventional poly(methyl methacrylate) (PMMA) method, and it exhibit similar color as that of as-grown MoS_2_ film, indicating that the MoS_2_ film was well transferred on the target substrate. Figure 1(c) shows AFM image of the transferred MoS_2_ film. The characteristic wrinkles of MoS_2_ are clearly visible. The inset in Fig. 1(c) shows the height profile along the white-dotted line in Fig. 1(c), and the thickness of MoS_2_ film is of around 0.85 nm, which is similar to the thickness of monolayer (ML) MoS_2_ (0.615 nm)^32^. It is worth noting that WS_2_ was successfully synthesized using another single liquid precursor that had been prepared by the dissolving of W(CO)_6_ in (CH_3_) _2_S_2_, instead of Mo(CO)_6_ (see Figure S2 in Supplementary Information).

A time evolution experiment was carried out to understand the growth behavior. Figure 1(d) shows scanning electron microscopy (SEM) images of as-grown MoS_2_ for 2.5, 5, 10, 15, 20, and 25 min, respectively. For 2.5 min growth, small ML MoS_2_ flakes were grown. Within 10 min, the area coverage of ML MoS_2_ increased up to 93%. After 15 min growth, the coverage of ML MoS_2_ is almost 94% with 6% few-layer (FL) MoS_2_. With a more prolonged growth time of 25 min, the portion of the FL MoS_2_ increased. Figure 1(e) displays the ML and FL coverage as a function of the growth time. The coverage of the ML MoS_2_ reached ~100% within 15 min and then gradually decreased with the growth time. On the other hand, the coverage of FL MoS_2_ increased from 15 min growth and reached almost 50% at 30 min of growth. This implies that ML MoS_2_ starts to grow at the initial stage as a bottom layer followed by the growth of FL MoS_2_ on top of monolayer MoS_2_, which is similar to the growth behavior of hexagonal boron nitride on Cu foils^33^.

**Figure 2:**
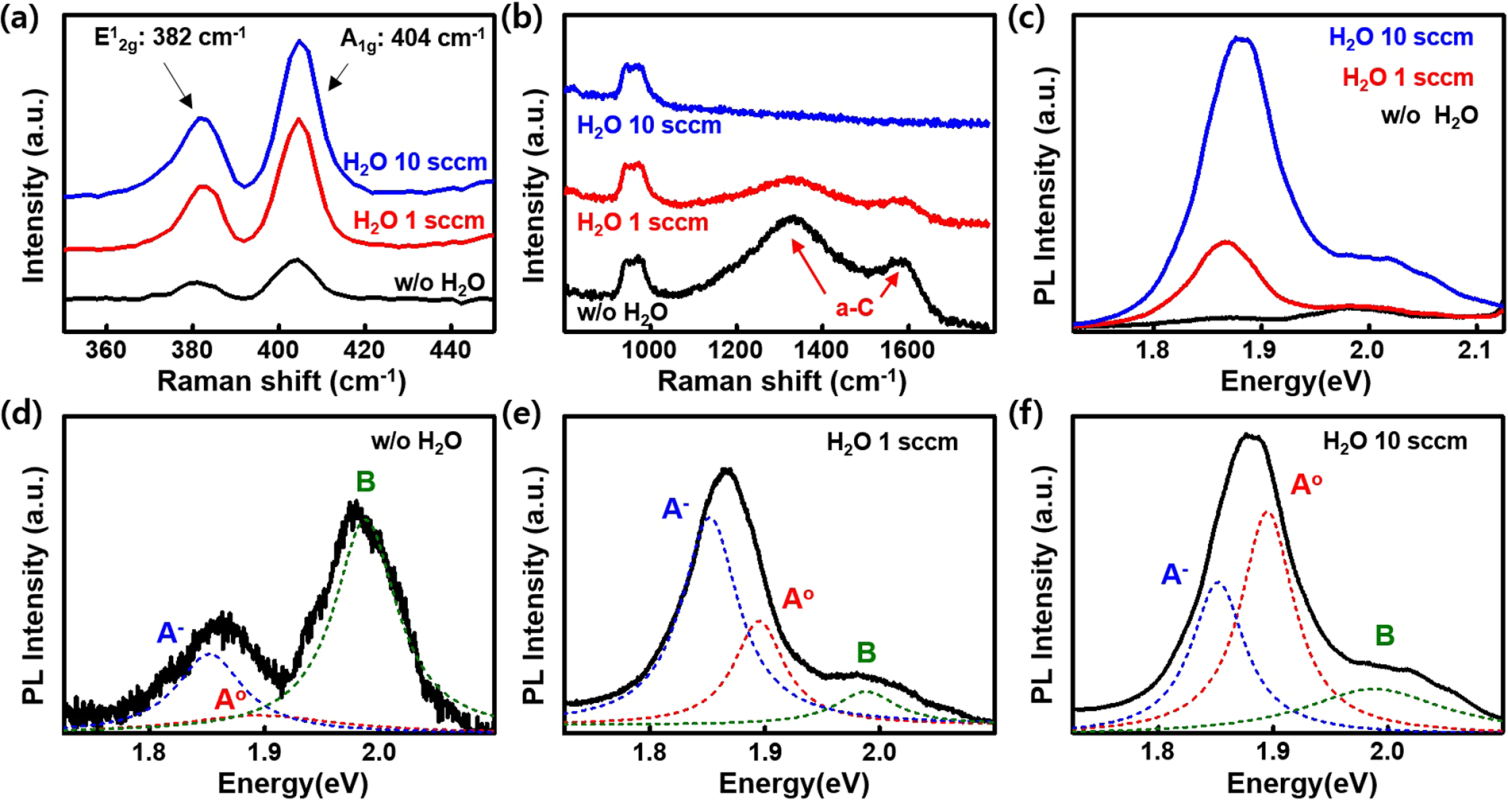
Effect of water supply I: the removal of a-C. (**a**,**b**) Raman and (**c**) PL spectra of MoS_2_ specimens with/without water supply (black: without (w/o) water, red: H_2_O 1 sccm, and blue: H_2_O 10 sccm, respectively). Each Raman spectrum for (**a**) and (**b**) display the presence of MoS_2_ and a-C, respectively. (**d**,**e**) Deconvoluted PL spectra of (**d**) w/o water, (**e**) H_2_O 1 sccm, and (**f**) H_2_O 10 sccm water with Lorentzian curves at 1.852 eV (blue, multiexciton, A^−^), 1.895 eV (red, neutral exciton, A°), and 1.988 eV (green, neutral exciton, B), respectively.

It is expected that carbon impurities will be present on the growth substrate since the precursor contains carbon. Therefore, the presence of carbon was confirmed via Raman spectroscopy. Figure 2(a,b) show the Raman spectra of MoS_2_ for different growth conditions: without water, with 1 sccm water, and with 10 sccm water. The peaks of E^1^_2g_ (382 cm^−1^) and A_1g_ (404 cm^−1^) originated from in-plane and out-of-plane phonon vibrations, respectively, are clearly seen for the sample grown without water supply (Fig. 2(a)). As expected, the peaks of the defect-related D-band near 1330 cm^−1^ and the graphite related G-band near 1580 cm^−1^ are detected in the Raman spectra, indicating that carbon impurities exist. We further confirmed the presence of amorphous carbon (a-C) on whole regions of the growth substrate via Raman mapping technique (see Figure S3 in Supplementary Information). Previous work showed that the a-C can be effectively eliminated with water, realizing the super growth of carbon nanotube forest^34^. Therefore, the water was introduced in this work as a weak oxidizer. The carbon atoms are eliminated by the following chemical reaction^34^.

1 $${\rm{C}}\,({\rm{s}})+{{\rm{H}}}_{2}{\rm{O}}\,({\rm{g}})={\rm{CO}}\,({\rm{g}})+{{\rm{H}}}_{2}\,({\rm{g}})$$

It is reported that the Gibbs free energy of Reaction 1 is changes from plus to minus at ~670 °C. This indicates that for Reaction 1 to be spontaneous, a minimum temperature of ~670 °C is required^35^. At 650 °C growth, the carbon impurities were not effectively removed (see Figure S4 in Supplementary Information). Reaction 1 shows that carbon monoxide and hydrogen molecule are evolved. With 1 sccm of water, the peaks of the D-band and G-band slightly decreased (Fig. 2(b)) whereas the peak intensities for E^1^_2g_ and A_1g_ increased (Fig. 2(a)). Eventually, the a-C is completely removed when 10 sccm water supply is used. We further confirmed the entire removal of a-C via Raman mapping (see Figure S5 in Supplementary Information). Typically, ML MoS_2_ exhibits a strong PL intensity due to the direct band transition nature^7^. Figure 2(c) shows the PL spectra of MoS_2_ with/without the water supply. While the PL intensity is very weak without a water supply, it gradually increases as a function of the flow rate of water. To clarify the change in the PL intensity, each PL spectrum was fitted with a Lorentzian curve, as shown in Fig. 2(d–f). In the absence of water, the intensity of the neutral exciton (A°) peak near 1.895 eV is very weak, whereas multiexciton (A^−^) near 1.852 eV and neutral exciton (B) near 1.988 eV are observed to be stronger^36^. However, those peaks are not strong when compared to those of water supply. As the water supply increases, the intensity of the A° exciton peak becomes more intense compared to those of other peaks, indicating that the overall PL intensity has increased. The change of PL intensity, such as PL quenching without water supply and the increase of A° exciton intensity with water supply might be related to the presence of a-C and molybdenum oxide. In the presence of a-C, the excited electron might be transferred to the conduction band of the conductive a-C, leading to PL quenching^37^. On the other hand, in the absence of a-C, a strong PL intensity is clearly observed. The presence of molybdenum oxides will be discussed later on, but the increase of the neutral exciton A° with the water supply might also be attributed to the undoping effect on MoS_2_ by the removal of the molybdenum oxide^38–40^. As a consequence, a-C is effectively removed via water oxidation. It is noted that the water supply promotes the generation of radicals by hydrolysis and hydrogenolysis reactions of precursors, expecting that small MoS_2_ flakes are grown^31^. Even though the flux of radicals increases in the presence of water, the amount of radicals which participate in the growth of MoS_2_ at high temperature decreases due to the higher desorption rate^41^. Therefore, a fair quality of MoS_2_ film within ~15 min is achieved.

**Figure 3:**
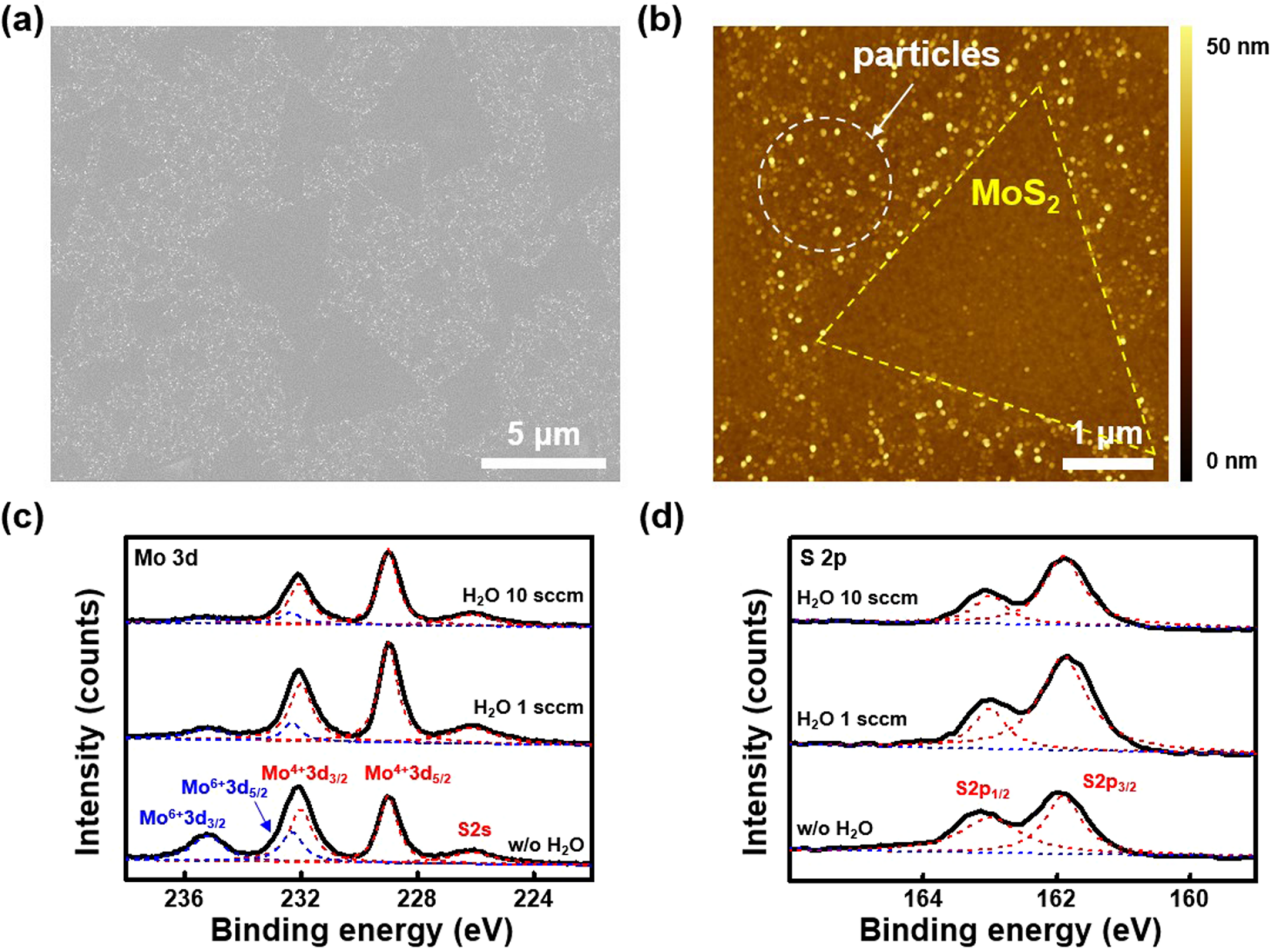
Effect of water supply II: removal of molybdenum oxides. (**a**) SEM, (**b**) AFM images of as-grown MoS_2_ flakes without the water supply. Unresolved particles are distributed on the whole region. (**c**,**d**) XPS core level spectra of (**c**) Mo 3d and (**d**) S 2p for three MoS_2_ specimens: without (w/o) water, H_2_O 1 sccm, and H_2_O 10 sccm, respectively. The characteristic peaks of Mo^4+^3d_3/2_, Mo^4+^3d_5/2_, S 2s, S 2p_1/2_, and S 2p_3/2_ correspond to MoS_2_ whereas the peaks of Mo^6+^3d_3/2_ and Mo^6+^3d_5/2_ correspond to molybdenum oxide.

Regarding the molybdenum oxide, we found unresolved particles from SEM and AFM images in the sample surface when water was not supplied, as shown in Fig. 3(a,b). Furthermore, it should be emphasized that full-coverage MoS_2_ film was not achieved even with growth time exceeding 2 hours with the presence of several particles. To elucidate the chemical composition of those particles, the samples were further analyzed via X-ray photoelectron microscopy (XPS) and transmission electron microscopy (TEM). Figure 3(c,d) show the XPS spectra of Mo 3d and S 2p core levels with/without water supply. Typically, bulk MoS_2_ shows three characteristic peaks for Mo^4+^3d_3/2_ (~232 eV), Mo^4+^3d_5/2_ (~229 eV), and S 2s (~226.13 eV) in the Mo 3d core level spectra and two peaks for S 2p_1/2_ and S 2p_3/2_ in the S 2p core level spectra^42^. Meanwhile the XPS spectra for the S 2p core level is similar regardless of the water supply, and the Mo 3d core level spectra changed due to the presence of water. When water was not supplied, four distinct peaks could be observed, and one peak near 235.2 eV disappeared gradually as the water supply increased (Fig. 3(c)). Gaussian curve fitting was used to assign the additional peaks in Mo 3d core level spectra to the Mo^6+^3d_3/2_ and Mo^6+^3d_5/2_ of molybdenum oxide^43^. It is not currently clear why molybdenum oxide formed, but those were eliminated when water was supplied. Hydrogen gases released in Reaction 1, reduces the molybdenum oxide as shown in Reaction 2, resulting in the generation of the molybdenum suboxide and water^44^.

2 $${{\rm{MoO}}}_{3}\,({\rm{s}})+{{\rm{H}}}_{2}\,({\rm{g}})={{\rm{MoO}}}_{3-{\rm{X}}}\,({\rm{g}})+{{\rm{H}}}_{2}{\rm{O}}\,({\rm{g}})$$

3 $${{\rm{MoO}}}_{3-{\rm{X}}}\,({\rm{g}})+(7-{\rm{x}})/2{\rm{S}}\,({\rm{g}})={{\rm{MoS}}}_{2}\,({\rm{s}})+(3-{\rm{x}})/2{{\rm{SO}}}_{2}\,({\rm{g}})$$

**Figure 4:**
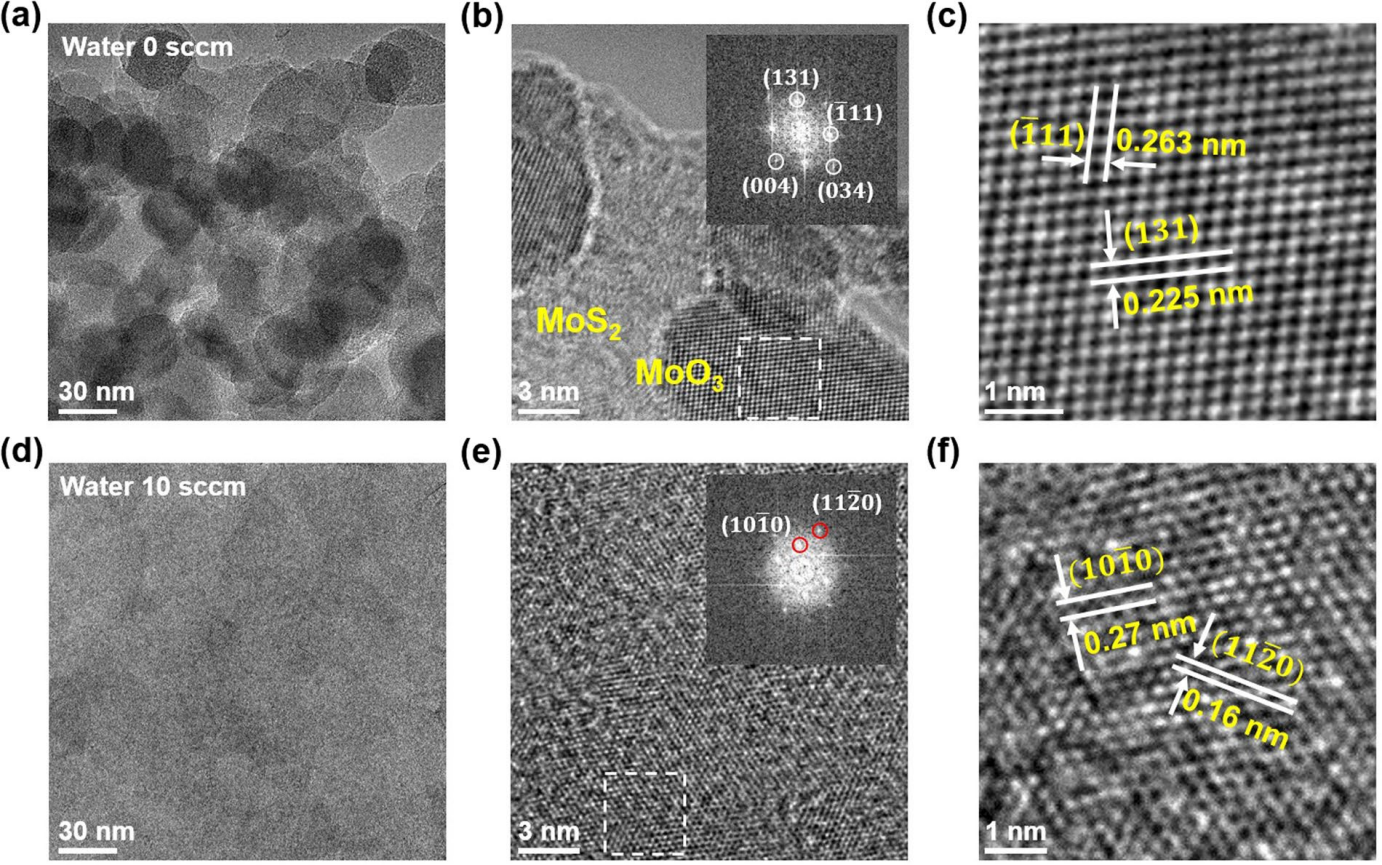
TEM analyses of unresolved particles and MoS_2_. (**a**–**f**) TEM images of MoS_2_ at different magnifications (**a**–**c**) with and (**d**–**f**) without water supply. The insets of (**b**) and (**e**) indicate the fast Fourier transform (FFT) patterns in the white-dashed box for each image. (**c**,**f**) Zoomed-in TEM images of white-dashed boxes in (**b**) and (**e**), respectively.

The generated suboxide participates in the formation of MoS_2_ via Reaction 3 ^20^. The removal of molybdenum oxide was further confirmed via TEM analysis. Figure 4 shows TEM images of MoS_2_ with/without water supply. Without water supply, molybdenum oxide particles are observed on the MoS_2_ film (Fig. 4(a,b)). In Fig. 4(b), the inset shows the fast Fourier transformation (FFT) of the white-dashed box. The parallelogram-shaped dots are assigned to the (131), (−111), (004), and (034) planes of MoO_3_, according to a previous reports^45, 46^. Figure 4(c) shows a high resolution TEM image of molybdenum oxide particles, and the parallelogram-shaped lattice structure is clearly identified. The value for d-spacing for the (−111) and (131) planes were obtained as 0.263 and 0.255 nm, which is in good agreement with the previous results obtained for molybdenum trioxide (MoO_3_)^45, 46^. Therefore, the particle is deduced to be MoO_3_ with an orthorhombic structure. With the water supply, such particles were not observed on the MoS_2_ film, as shown in Fig. 4(d,e). The hexagonal-shaped FFT pattern in the inset of Fig. 4(e) is obtained from the white-dashed box in Fig. 4(e). The hexagonal dots are assigned to the (10–10) and (11–20) planes of MoS_2_, according to a previous report^47^. The high-resolution TEM image in Fig. 4(f) shows the apparently hexagonal structure of MoS_2_. The d-spacings for the (10–10) and (11–20) planes are 0.27 nm and 0.16 nm, respectively. Those values match well with previously reported values^20^, indicating that the MoS_2_ film has been successfully synthesized.

**Figure 5:**
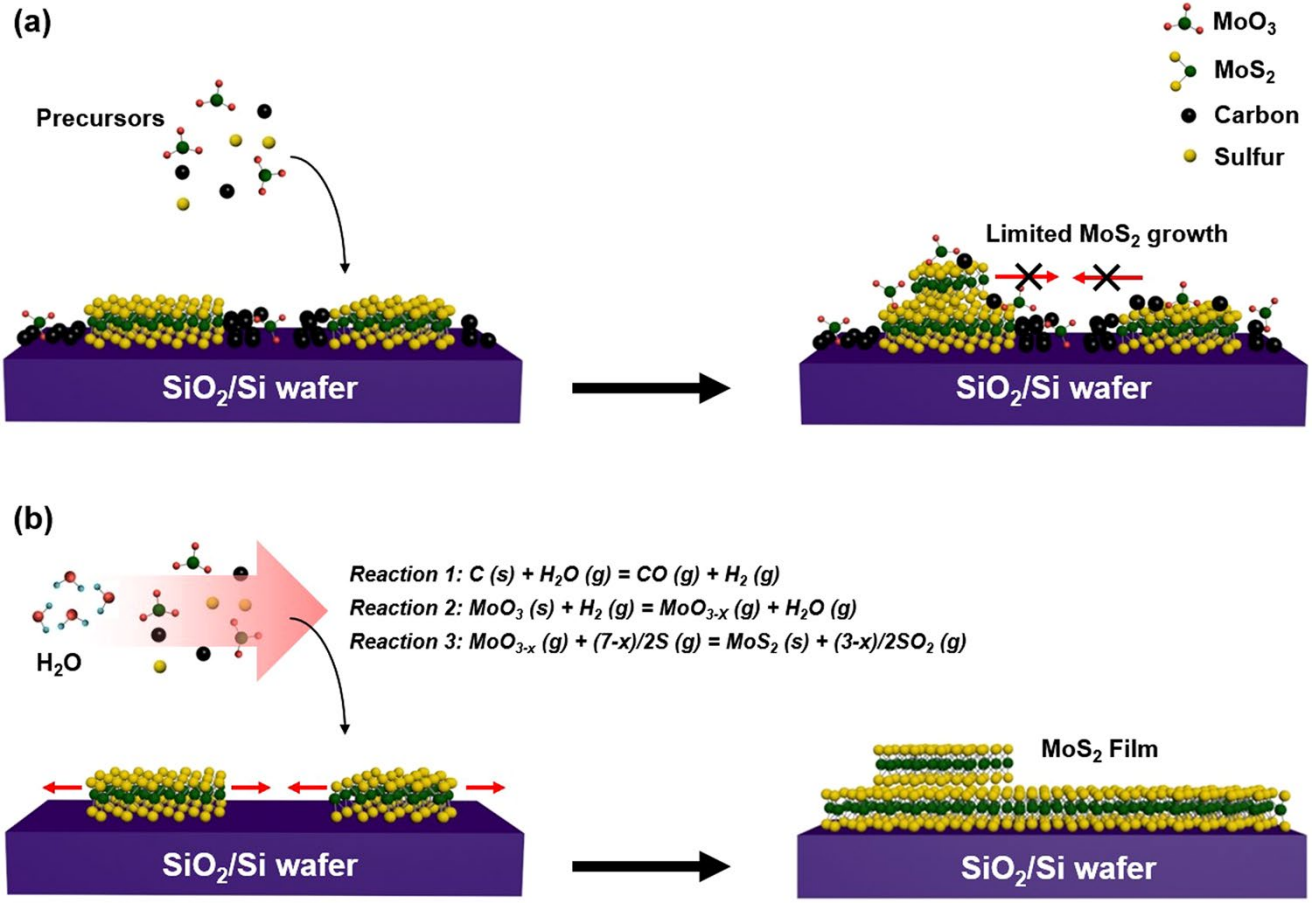
Different growth behavior. (**a**,**b**) Schematic illustration of MoS_2_ growth (**a**) without and (**b**) with water supply. Without water supply, unwanted impurities such as MoO_3_ and a-Cs inhibit the completed MoS_2_ film. In the presence of water, the full-covered MoS_2_ film is grown through Reactions 1–3.

Figure 5 illustrates the growth mechanism for MoS_2_ with/without water supply. In the absence of water, various kinds of molybdenum oxides and a-Cs are easily deposited on the growth substrate during growth, resulting in the formation of MoO_3_ particles and a-Cs. These unwanted impurities limit the evolution of continuous the MoS_2_ film (Fig. 5(a)). In contrast, the water supply during growth can lead to effective removal of a-C via Reaction 1. In addition, the hydrogen gases stemming from Reaction 1 plays an important role in removing the impurities and enhancing the lateral growth of the MoS_2_. Firstly, molybdenum oxide is reduced to molybdenum suboxide by the hydrogen, releasing water and carbon monoxide via Reaction 1. Secondly, the reduced molybdenum suboxides participate in the lateral growth of the MoS_2_ film via Reaction 3, resulting in full coverage of the MoS_2_ film without any other impurities (Fig. 5(b)). As a consequence, MoS_2_ films can be synthesized with water oxidation of a-C and reduction of molybdenum oxide impurities.

## Conclusions

We have synthesized MoS_2_ films using a single liquid precursor made by dissolving Mo(CO)_6_ in (CH_3_)_2_S_2_. The liquid precursor consists of Mo ions, Mo compounds and sulfur compounds. Unlike with a solid precursor, full-coverage MoS_2_ can be obtained simply by increasing the growth time. We found the introduction of water to be important in removing unwanted impurities, such a-C and molybdenum oxides from the MoS_2_ film. Carbon was removed by water oxidation in Reaction 1: C (s) + H_2_O (g) = CO (g) + H_2_ (g), and the hydrogen gas stemming from Reaction 1 helps effectively remove the molybdenum oxide. As a consequence, an impurity-free MoS_2_ film was grown with the assistance of water. Our approach does not only open the use of organic liquid precursors in the synthesis of MoS_2_ but also advances the synthesis of other s-TMdCs.

## Methods

### Substrate preparation

20 mm × 20 mm Si substrate with a 300 nm thick SiO_2_ layer was kept in Piranha solution to remove the organic residues and produce a hydrophilic surface. After rinsing and drying the substrate, 0.01 wt% PTAS solution was coated on the substrate as a seeding promoter via spin-coating. The substrate was placed on the center of a 5 cm × 5 cm quartz plate.

### Preparation of the single liquid precursor

0.5 g of molybdenum hexacarbonyl (Mo(CO)_6_, >99.9%, Sigma Aldrich) powder was dissolved in 50 mL of dimethyl disulfide (CH_3_SSCH_3_, >99%, Sigma Aldrich). The mixture was kept in a quartz bubbler. To prevent the agglomeration, the solution was stirred with a magnetic bar on a home-made stirring system.

### Growth of the MoS_2_ film

A furnace and a 2-inch quartz tube were connected and equipped with high-purity (99.999%) argon, hydrogen gas, and two bubblers (liquid precursor and water). The growth substrate was loaded at the center of the quartz tube. Prior to growth, the system was purged using argon at 500 sccm for 10 minutes. The quartz tube was rapidly heated up to 750 °C for 8 min in a preheated furnace. During growth, the temperature was maintained with 5 sccm of precursor and 10 sccm of water flow for 15 minutes. After growth, the quartz tube was rapidly cooled down to room temperature by taking the quartz out of the furnace. 350 sccm of argon flow rate at atmospheric pressure was maintained throughout the entire growth process.

### Characterization

The liquid precursor mixture was analyzed via liquid chromatography mass spectrometry (XEVO TQ-S, Waters). The surface morphologies of MoS_2_ were characterized via optical microscopy (Nikon LV-IM, Nikon), scanning electron microscopy (JSM-7100F, JEOL), and atomic force microscopy (N8-NEOS, Bruker). To identify phonon vibration and photoluminescence of sample, a micro-Raman system (XperRam100, Nanobase) was used with a 532-nm laser. The laser power was kept at 0.1 mW to avoid damaging the sample. The chemical composition of MoS_2_ was analyzed via X-ray photoelectron spectroscopy (K-alpha, Thermo fisher scientific), and the atomic structures of MoS_2_ and the molybdenum oxide particles were characterized by transmission electron microscopy (Tecnai, FEI). The acceleration voltage was 200 kV during the TEM measurement. Prior to TEM measurement, the MoS_2_ were transferred on a TEM grid using conventional PMMA transfer^48^.

## Electronic supplementary material

Supplementary Information
