# Supplementary material for: Water-Assisted Synthesis of Molybdenum Disulfide Film with Single Organic Liquid Precursor
Source: Sci Rep. 2017 May 16;7:1983. doi: 10.1038/s41598-017-02228-8 (PMC5434056; doi:10.1038/s41598-017-02228-8)
Supplement: Supplementary file 1 — Supplementary Information [file 41598_2017_2228_MOESM1_ESM.doc]

**Supplementary Information**

# Water-Assisted Synthesis of Molybdenum Disulfide Film with Single Organic Liquid Precursor by Soo Ho Choi, Boandoh Stephen, Ji-Hoon Park, Joo Song Lee, Soo Min Kim, Woochul Yang and Ki Kang Kim


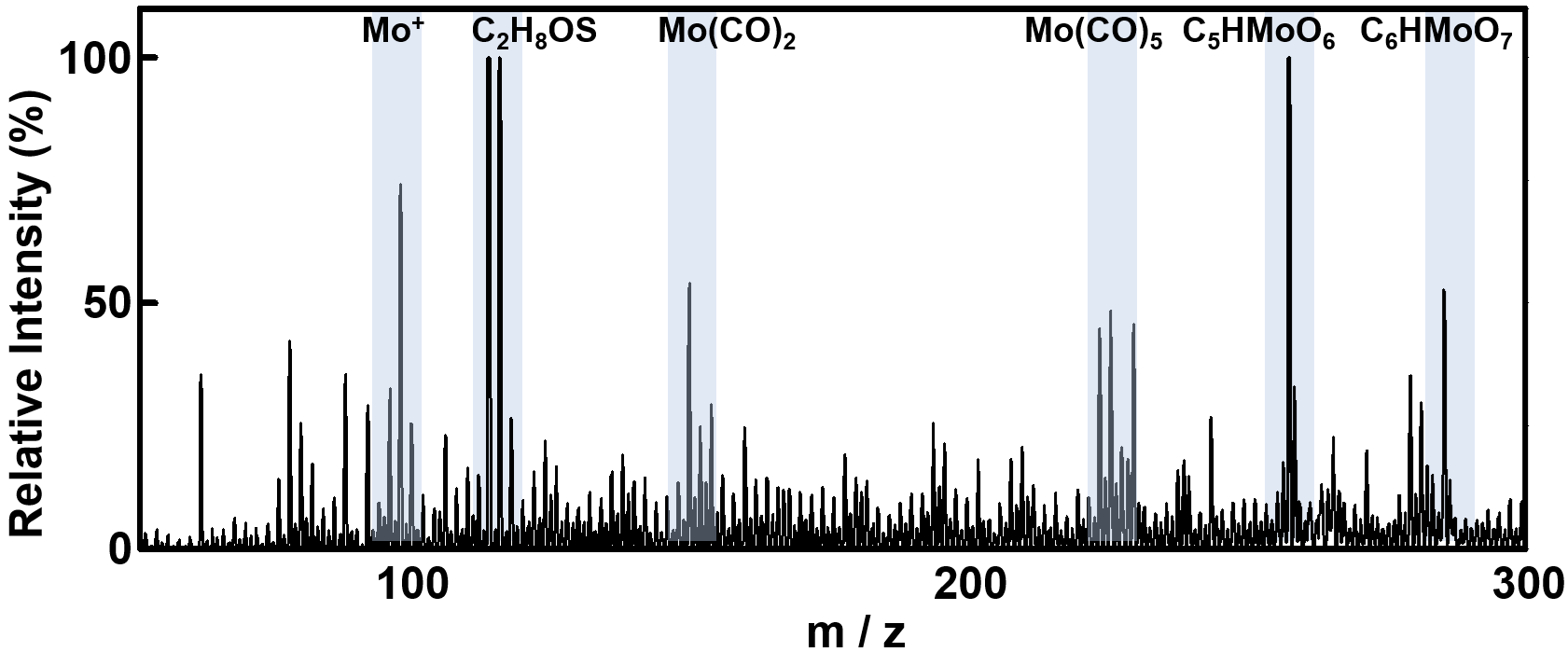


**Figure S1. Mass spectrum of the liquid precursor for MoS_2_ growth.** Mo and S chemicals were present, including Mo ions, C_2_H_8_OS, Mo(CO)_2_, Mo(CO)_5_, C_5_HMoO_6_, and C_6_HMoO_7_. Unfortunately, dimethyl disulfide was not detected due to the detection limit.

**Supplementary Information**

# Water-Assisted Synthesis of Molybdenum Disulfide Film with Single Organic Liquid Precursor by Soo Ho Choi, Boandoh Stephen, Ji-Hoon Park, Joo Song Lee, Soo Min Kim, Woochul Yang and Ki Kang Kim


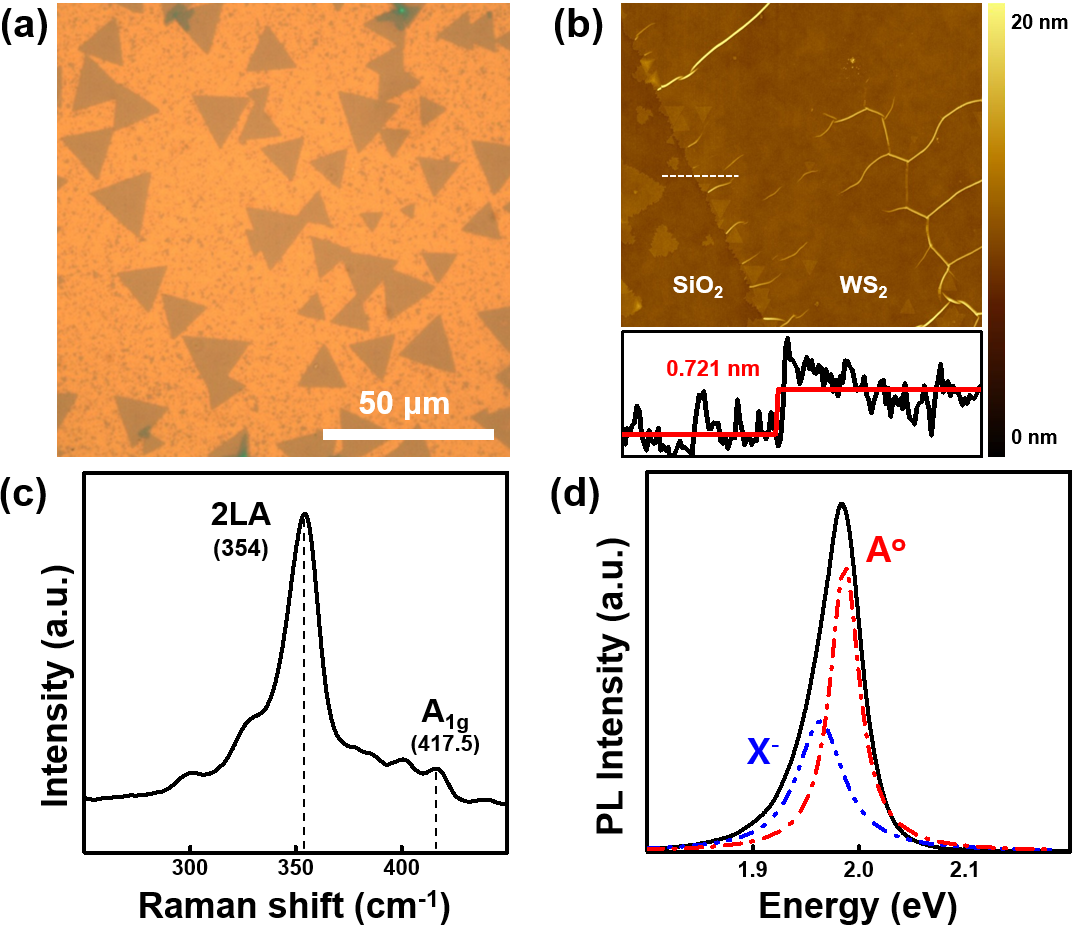


**Figure S2. Growth of WS_2_ flakes using a liquid precursor.** (**a**) Optical image of the as-grown WS_2_ flakes. (**b**) AFM image and height profile of WS_2_ flakes transferred on the SiO_2_/Si substrate. The thickness of the WS_2_ flake was about 0.72 nm, indicating monolayer WS_2_. (**c**) Raman and (**d**) PL spectra of as-grown WS_2_ flake. The characteristic phonon modes of 2LA and A_1g_ are clearly observed in the Raman spectrum, and the strong PL intensity of A^o^ was detected in the PL spectrum.

**Supplementary Information**

# Water-Assisted Synthesis of Molybdenum Disulfide Film with Single Organic Liquid Precursor by Soo Ho Choi, Boandoh Stephen, Ji-Hoon Park, Joo Song Lee, Soo Min Kim, Woochul Yang and Ki Kang Kim


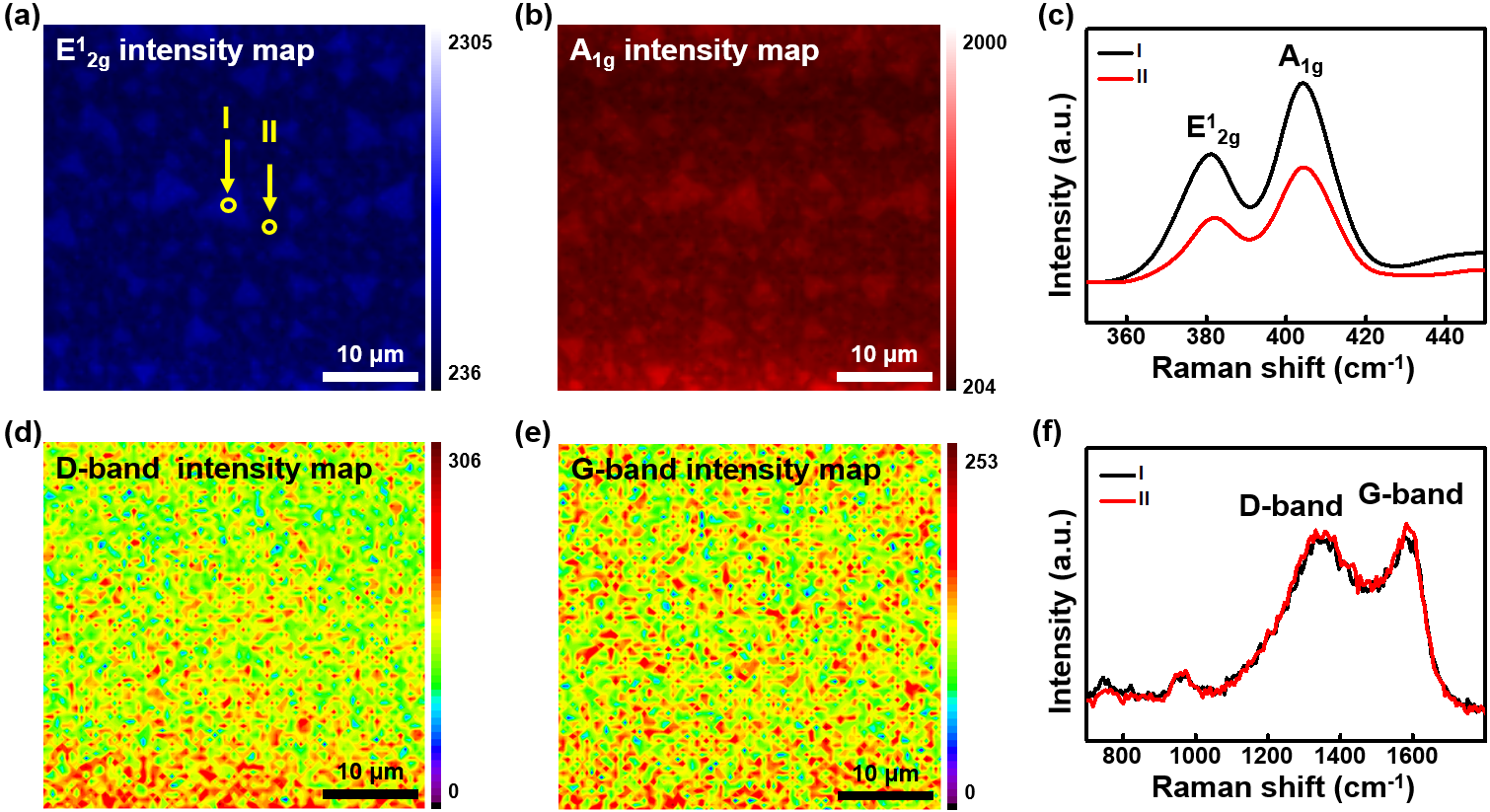


**Figure S3. Raman mapping images of as-grown MoS_2_ without water supply.** (**a,b,d,e**) Raman intensity map images of the sample at (**a**) E^1^_2g_, (**b**) A_1g_, (**d**) D-band, and (**e**) G-band, respectively. (**c,f**) Representative Raman spectra extracted from I and II in (a).

**Supplementary Information**

# Water-Assisted Synthesis of Molybdenum Disulfide Film with Single Organic Liquid Precursor by Soo Ho Choi, Boandoh Stephen, Ji-Hoon Park, Joo Song Lee, Soo Min Kim, Woochul Yang and Ki Kang Kim


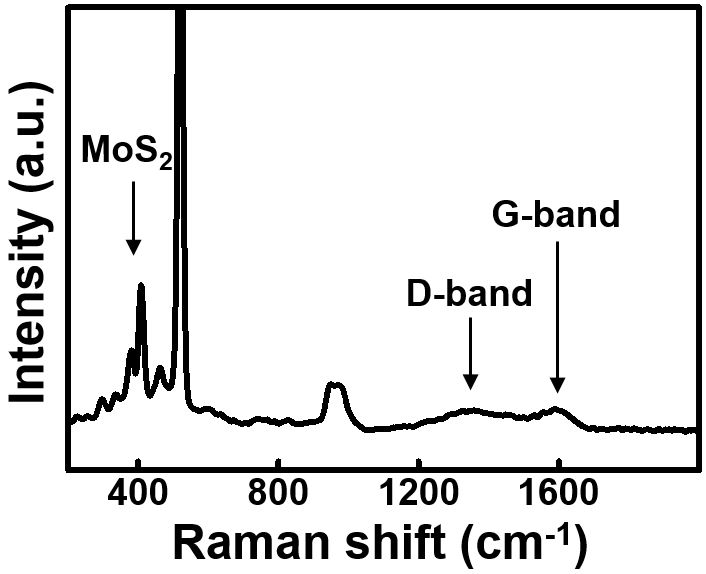


**Figure S4. Representative Raman spectrum of the MoS_2_ flakes grown at 650 ^o^C with water supply.** The a-Cs were not effectively removed.

**Supplementary Information**

# Water-Assisted Synthesis of Molybdenum Disulfide Film with Single Organic Liquid Precursor by Soo Ho Choi, Boandoh Stephen, Ji-Hoon Park, Joo Song Lee, Soo Min Kim, Woochul Yang and Ki Kang Kim


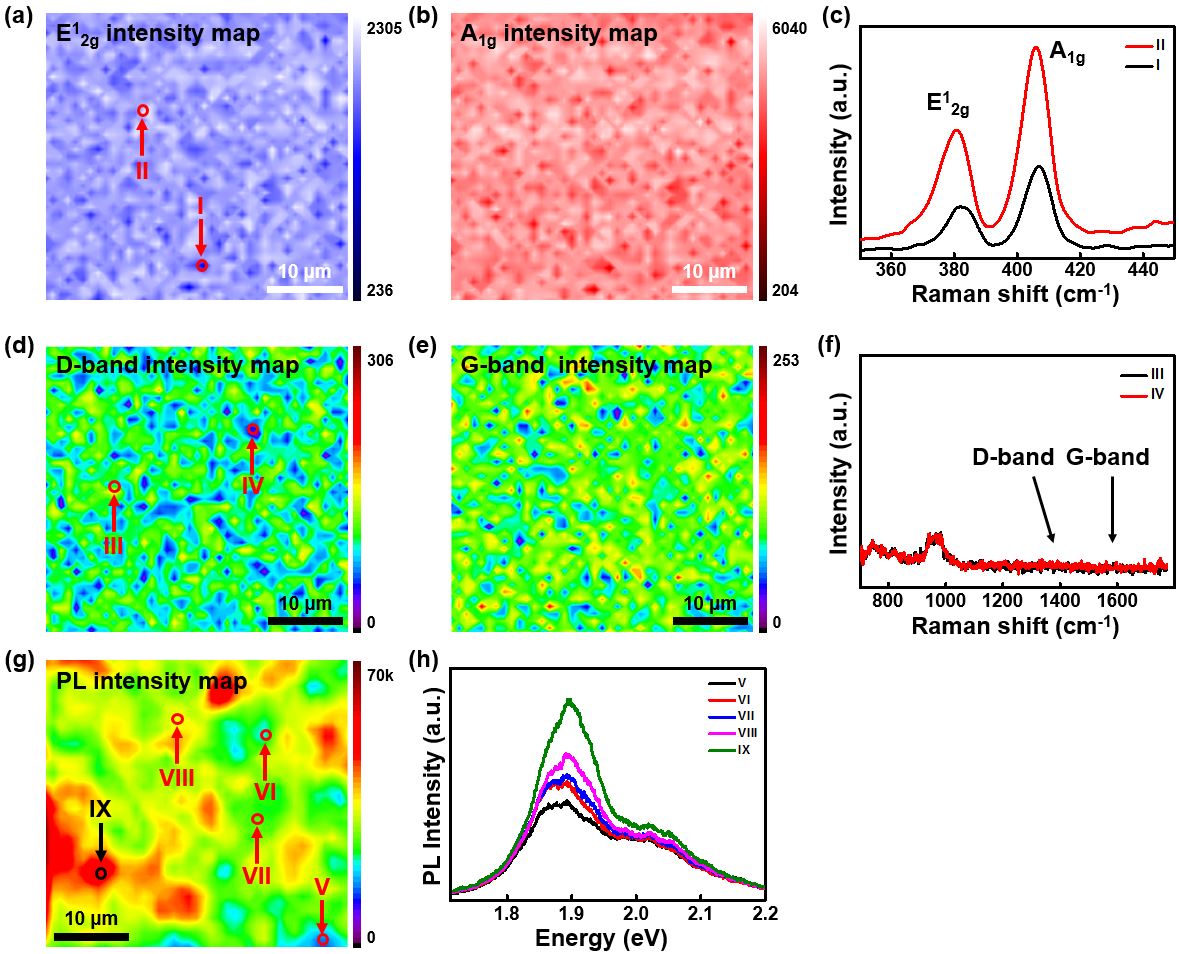


**Figure S5. Raman and PL mapping images of as-grown MoS_2_ with water supply.** (**a,b,d,e**) Raman intensity map images of the sample at (**a**) E^1^_2g_, (**b**) A_1g_, (**d**) D-band, and (**e**) G-band, respectively. (**c,f**) Representative Raman spectra extracted from I, II, III, and IV in (a,d). (**g**) PL intensity map image of as-grown MoS_2_ film with water supply. (**h**) PL spectra extracted from V, VI, VII, VIII, and XI in (g).
